# Study on the effect of compound cultivation on the growth feature and active ingredients content of *Salvia miltiorrhiza*

**DOI:** 10.3389/fpls.2023.1238896

**Published:** 2024-01-18

**Authors:** Luyi Zhang, Shan Tao, Yifan Zhang, Yanmei Yang, Fang Peng, Hailang Liao, Changqing Mao, Xiufu Wan, Yu Wu, Zhengjun Xu, Chao Zhang

**Affiliations:** ^1^ Industial Crop Research Institute, Sichuan Academy of Agricultural Sciences, Chengdu, Sichuan, China; ^2^ Crop Ecophysiology and Cultivation Key Laboratory of Sichuan Province, Sichuan Agricultural University, Chengdu, Sichuan, China; ^3^ State Key Laboratory of Dao-di Herbs, China Academy of Chinese Medical Sciences, Beijing, China

**Keywords:** *Salvia miltiorrhiza*, 16S rRNA, complex cultivation, inter-root secretions, microbial community

## Abstract

We investigated the effects of the complex cultivation of *Salvia miltiorrhiza* on microbial communities, secretions, yield, and active ingredients, and the mechanism of action between microbial communities, secretions, and *S. miltiorrhiza* growth and development. Neither maize nor soybean was suitable to grow with *S. miltiorrhiza*, but sesame significantly increased salvinone content, the active ingredient of *S. miltiorrhiza*, and Tanshinone IIA, Tanshinone I, and Cryptotanshinone increased by 27.06%, 22.76%, and 26.41%, respectively, which increased the abundance and number of microbial communities in *S. miltiorrhiza* roots. 16S rRNA results showed that the most abundant bacterial phyla were Proteobacteria and Acidobacteriota, and their number increased with compound planting of sesame and *S. miltiorrhiza*. *Salvia* inter-root secretions affected the microbial community and *Salvia* growth and development, and lipids and lipid-like molecules significantly reduced *Salvia* yield and active ingredients. Overall, different plant secretions can lead to differences in the natural environment and *Salvia* root growth and development, and the composite planting of sesame with *Salvia* can improve inter-root microbial communities, enhance *Salvia* quality, and make fuller use of land resources.

## 1 Introduction

*Salvia miltiorrhiza* Bge. is a perennial herb in the family Labiatae, whose roots and rhizomes contain active ingredients (tanshinones and tannins) with therapeutic effects on diseases such as cardiovascular diseases, diabetes, and liver diseases (Liu et al., 2020c; Jiang et al., 2022a). *S. miltiorrhiza* is a medicinal herb widely grown in bulk in Henan, Sichuan, Shandong, and Shaanxi (Wang et al., 2019). However, as adoption of highly efficient monocultures can lead to a decrease in quality and yield, measures such as composite cultivation are needed to promote sustainable development (Liu et al., 2020a).

High-efficiency monocultures usually lead to disturbances in soil physicochemical properties, reduced microbial community diversity, and toxin accumulation (Xin-hui et al., 2015). Recently, compound cropping has become an important research direction to solve continuous crop challenges. Compound cropping can increase plant species diversity, improve soil physicochemical properties, and enhance microbial community abundance by growing two or more crops simultaneously (Tang et al., 2014; Maitra, 2019; Domeignoz-Horta et al., 2020; Tang et al., 2021). Compound cropping can, therefore, increase agroecosystem diversity and promote sustainable agriculture (Stomph et al., 2020). Studies have shown that complex planting can modify plant root natural environments (Wang et al., 2019), and stimulate the production of unique inter-root secretions (Casper and Castelli, 2007), which regulate plant growth and promote nutrient uptake, attract specific probiotic bacteria to form plant-soil feedbacks (PSF) (Neumann and Romheld, 2003; Bais et al., 2006), and influence microbial community formation (Sasse et al., 2018).

The beneficial effects of intercropping in complex cropping systems, such as those involving cereals and legumes, have been extensively researched (Hsiao et al., 2019). Scholars have found that maize inter-root secretions contribute to enhanced reciprocity with legumes and promote nitrogen fixation by legumes (Jiang et al., 2022b). Hu demonstrated that maize inter-root secretions promote flavonoids synthesis in faba beans (Hu et al., 2021). However, not all plant species are conducive to a good inter-root microecological environment. For example, the combination of maize and *S. miltiorrhiza* was detrimental to growth and yield of the latter (Deng et al., 2017). Therefore, it is crucial to investigate suitable plants for successful *Salvia* complex cultivation.

Lei et al. (2018) found that different plants have a significant effect on *S. miltiorrhiza* growth and development, with pepper showing the greatest improvement in yield and quality. *S. miltiorrhiza* dry weight increased by 12.52%, salvianolic acid B increased by 10.25%, and tanshinone compound content significantly increased by 58.91%. Similarly, Liu et al. (2018) investigated the intercropping effects of mint, perilla, and alfalfa with *S. miltiorrhiza*. They found that they produced the highest weight gain and increased content of *S. miltiorrhiza* active ingredients. Moreover, the content of lipid-soluble components cryptotanshinone, tanshinone I, and tanshinone IIA in *S. miltiorrhiza* significantly increased after intercropping treatment, with the highest tanshinone content of 1.08% in *S. miltiorrhiza* roots after *S. miltiorrhiza*–mint intercropping, which was 163.41% higher than after monocropping. However, there has been no suitable grain and oil crop identified for intercropping with *S. miltiorrhiza*, and little research has been conducted on the effects of different plants on *S. miltiorrhiza* inter-root microbial community structure and abundance. Further research is needed to explore suitable crops and the underlying mechanisms of action.

To address these shortcomings, we investigated the effects of three crop plants on *S. miltiorrhiza* inter-root microecology and growth and development. The study designed pot experiments using macrogenomics and metabolomics to analyze *S. miltiorrhiza* inter-root microecology and investigate its responses to different plant secretions, including changes in soil physicochemical properties, bacterial abundance, inter-root soil community composition, and metabolite production. To confirm our results, we conducted field plot experiments. We hypothesized that various plants would lead to different *Salvia* inter-root microbial populations and community structures, affect its root morphology, and alter its inter-root secretions.

## 2 Materials and methods

### 2.1 Experimental site

The experiments were conducted at the Industrial Crop Research Institute, Sichuan Academy of Agricultural Sciences, Qingbaijiang District, Chengdu City, Sichuan Province, China (30°42′N, 104°19′E), where average annual temperature was 18°C, and average annual precipitation was 558.1 mm (local weather bureau data). *Salvia* seedlings and maize, sesame, and soybean varieties were provided by the Institute of Economic Crop Breeding and Cultivation, Sichuan Academy of Agricultural Sciences. Basic Soil Properties: Potted Plants, pondus hydrogenii = 6.7, organic matter = 21.90 g/kg, available P = 43.76 mg/kg, available K = 115.50 mg/kg, total nitrogen = 1.48 g/kg; Field, potential of hydrogen = 6.0, organic matter = 19.78 g/kg, available P = 26.75 mg/kg, available K = 188.27 mg/kg, total nitrogen = 1.48 g/kg.

### 2.2 Pot experiments and field experiments

**Figure 1:**
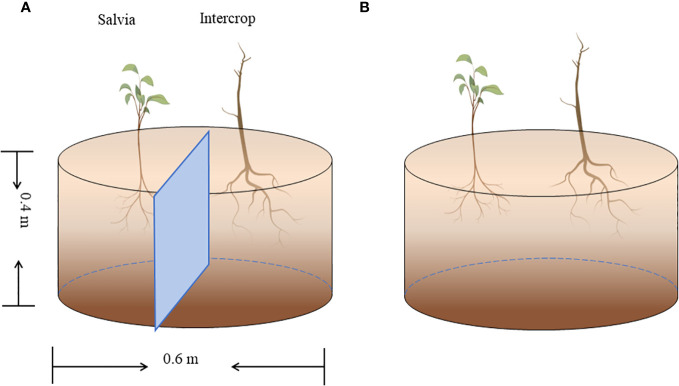
Pot experiment (*n* = 5). **(A)** Complete isolation of *Salvia* and other crops with plastic film, and **(B)** no isolation of *Salvia* and other crops.

Pot experiments were set up in six planting combinations: maize *+ Salvia* completely isolated (*Salvia*|Maize), maize *+ Salvia* without isolation (*Salvia*Maize), soybean *+ Salvia* completely isolated (*Salvia*|Soybean), soybean *+ Salvia* without isolation (*Salvia*Soybean), sesame *+ Salvia* completely isolated (*Salvia*|Sesame), and sesame *+ Salvia* without isolation (*Salvia*Sesame). *S. miltiorrhiza* was propagated from fresh root segments (1.3–1.8 cm in diameter) selected from seedlings dug in January 2022, and transplanted to pots and the field after seedlings were raised in the greenhouse. The pot experiment used 0.4-m-high, 0.6-m-diameter breeding bags, including two isolation treatments and was conducted in a randomized design (**Figure 1**): ① plastic film isolation where the two crops were completely separated, with no inter-root intercropping; and ② without any isolation, where the two crops can communicate openly between the roots, with inter-root secretions interacting with each other, and where inter-root intercropping is obvious. Five replicates of each treatment were made.

**Figure 2:**
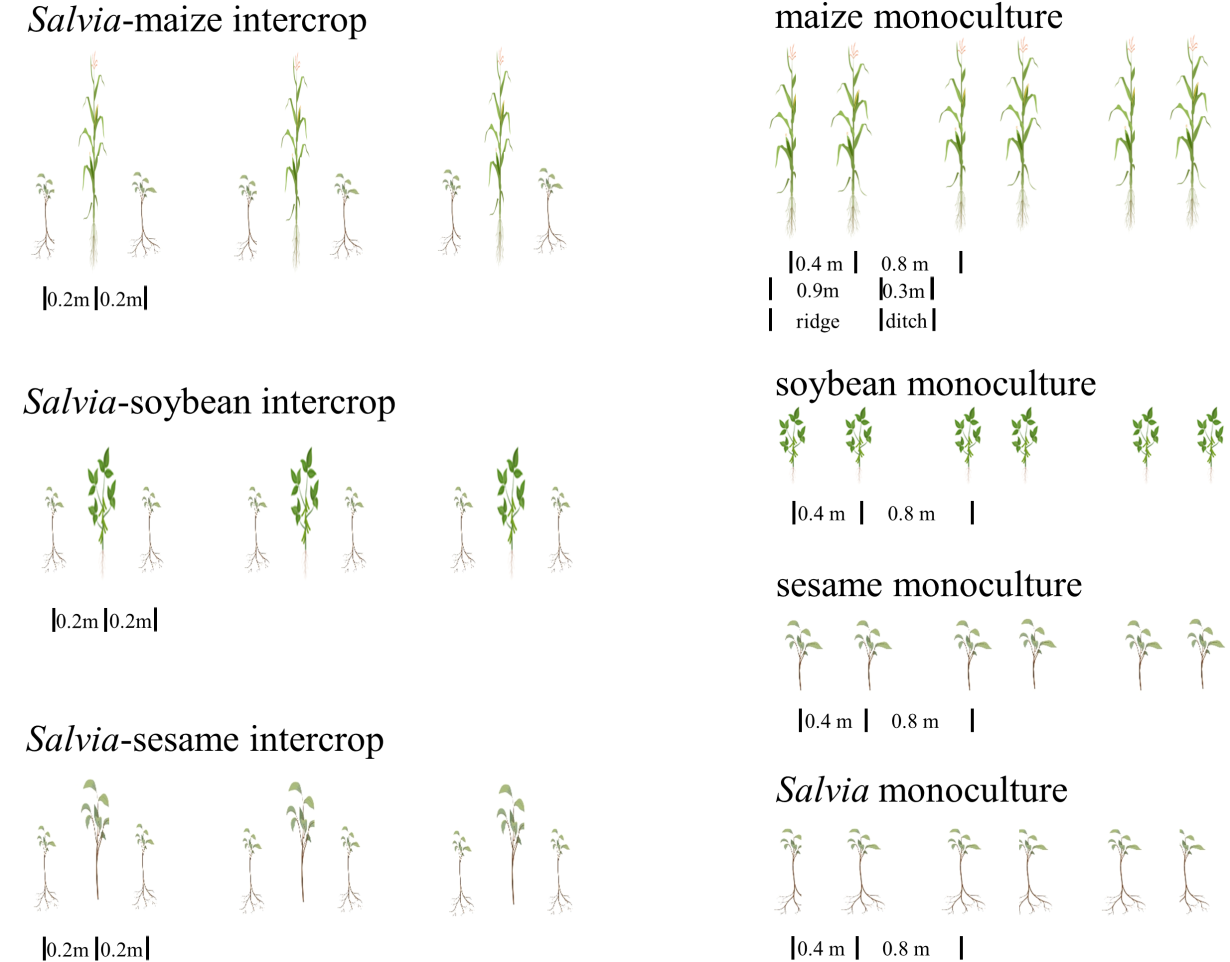
Layout of the fifield experiment (n = 3).

The field experiments were conducted in a randomized design (**Figure 2**), including soybean monoculture (soybeanm, 0.25 m spacing), maize monoculture (maizem, 0.4 m spacing), sesame monoculture (sesamem, 0.25 m spacing), *Salvia* monoculture (*salvia*m, 0.25 m spacing), *Salvia*-maize intercrop (*Salvia* + maize), *Salvia*-soybean intercrop (*Salvia* + soybean), and *Salvia*-sesame intercrop (*Salvia* + sesame). The size of each experimental area was 3.6 × 5 m, and each treatment was replicated three times.

### 2.3 Inter-root soil and general soil sampling

Soil samples were randomly taken from the field and pots at the time of sowing in March, and later mixed into composite samples that were naturally air-dried and stored in an oven for testing. Sampling was carried out in December 2022 on normal soil and inter-rhizosphere soil (soil close to plant roots and usually containing higher microbial populations than the surrounding normal soil) (Fierer, 2017). Inter-root soil (still attached to the roots) was shaken off gently and stored at −80°C for microbial community and inter-root metabolite analysis (Zhao et al., 2021). Simultaneously, soil around *S. miltiorrhiza* plants was collected, plant roots and stones were removed, and the soil was put into a self-sealing bag, and then it was dried and ground naturally, passed through a 60-mesh sieve and set aside for soil physical and chemical property testing.

### 2.4 Determination of soil physical and chemical properties

Soil potential of hydrogen (pH) was determined by the point method (HJ 962-2018), where soil/water is mixed at 1/2.5 (w/v), stirred vigorously for 2 min, and left for 30 min, and then pH was measured with a pH meter (PHS-SE) (Pang et al., 2021). Soil organic matter (SOM) was determined using the Walkley–Black method, which oxidizes SOM by H_2_SO_4_ and K_2_Cr_2_O_7_, followed by titration using FeSO_4_ (Fu et al., 2015). Soil active phosphorus (available P) was measured by a spectrophotometer at 700 nm (NY/T 1121.7-2014) (Liu et al., 2018). Soil available potassium (available K) was determined by a flame photometer after extraction by 1 mol/L ammonium acetate (Saber et al., 1973). Soil total nitrogen was determined by the Kelvin digestion-constant distillation–titration method, followed by digestion using an automatic Kjeldahl nitrogen tester (Hennen K1100) (Wang et al., 2018).

### 2.5 Microbial community structure and diversity analysis

A Soil DNA Kit was used to isolate soil microbial DNA from 5-g soil samples. The V4 hypervariable region of the bacterial gene was amplified with PCR using primers F (ACTCCTACGGGAGGCAGCA) and R (GGACTACHVGGGTWTCTAAT). After PCR processing, sequencing was conducted using an Illumina NovaSeq 6000. The raw image data files were converted to raw Sequenced Reads by Base Calling analysis. Sequenced data were filtered for connectors and low-quality using FASTP and Trimmomatic for Raw Reads to obtain high-quality valid sequences (Berg et al., 2015). Finally, the sequences were clustered with USEARCH (Edgar, 2013) (version 10.0) at the 97% similarity level, and OTUs were filtered using 0.005% of the total number of sequences sequenced as a threshold (Bokulich et al., 2013).

### 2.6 Salvia inter-root metabolites

To analyze metabolites in inter-rhizosphere soil, a 50-mg sample was weighed and mixed with 1,000 μL of extraction solution containing internal standard. The mixture was then ground with steel beads and sonicated for 10 min in an ice water bath, followed by centrifugation, and the supernatant was carefully collected for testing. Metabolomics analysis was conducted using a liquid mass spectrometry system composed of a Waters Acquity I-Class PLUS UPLC tandem and a Waters Xevo G2-XS QTof high-resolution mass spectrometer (Acquity UPLC HSS T3, 1.8 μm 2.1*100 mm). Positive ionization mode: mobile phase A: 0.1% formic acid aqueous solution; mobile phase B: 0.1% formic acid acetonitrile; negative ionization mode: mobile phase A: 0.1% formic acid aqueous solution; mobile phase B: 0.1% formic acid acetonitrile. The Waters Xevo G2-XS QTof high-resolution mass spectrometer is capable of primary and secondary mass spectrometry data acquisition in MSe mode under acquisition software (MassLynx V4.2, Waters) control. In each data acquisition cycle, dual-channel data acquisition is possible for both low and high collision energies. Low collision energy was 2 V, the high collision energy range was 10–40 V, and scan frequency was 0.2 s for one mass spectral map. ESI ion source parameters were as follows: capillary voltage: 2,000 V (positive ion mode) or −1,500 V (negative ion mode); cone hole voltage: 30 V; ion source temperature: 150°C; desolvent gas temperature: 500°C; backblast gas flow rate: 50 L/h; desolvent gas flow rate: 800 L/h. Raw data collected by MassLynx V4.2 were processed by Progenesis QI software for peak extraction, alignment, and other data processing operations.

### 2.7 Salvia root morphology and active ingredients (tanshinones and tannins)

*S. miltiorrhiza* was harvested in December, its inter-root morphology was investigated, and its yield was measured. The number of branches, fresh weight, and dry weight of individual roots were also measured at harvest, along with root length (measured from the base of the stem) and diameter (the largest *S. miltiorrhiza* diameter) (Lei et al., 2018). After harvesting, the rhizome was dried, powdered, sieved (60 mesh), and stored to determine active ingredients. Intercrops were harvested in June and July.

*S. miltiorrhiza* active ingredients (tanshinones and tannins) were determined using high-performance liquid chromatography. Total tanshinones and tannic acid B were determined according to the Chinese Pharmacopoeia 2015 edition, wherein tanshinones (Tanshinone IIA, Cryptotanshinone, and Tanshinone I) shall not be<0.25% in total and tanshinolic acid B shall not be<3.0%. Chromatographic analysis was performed with high-performance liquid chromatography equipment (Agilent 1260 series, USA) on a ZORBAXSB-C18 (4:6 mm × 250 mm, 5 μm) column at a temperature of 20°C. For salvianolic acid B determination, the mobile phase consisted of acetonitrile (A) and 0.1% phosphoric acid aqueous solution (B) in gradient elution mode for 20 min at 90% A with a flow rate of 1.0 mL min^−1^ and an injection volume of 10 μL at a detection wavelength of 286 nm. The mobile phase was composed of acetonitrile (A) and 0.02% phosphoric acid aqueous solution (B): 0–6 min, 0%–61% A; 6–20 min, 61%–90% A; 20–20.5 min, 90%–61% A; 20.5–25 min, 61% A, with a flow rate of 0.8 mL min^−1^, a sample volume of 10 μL, and a detection wavelength of 270 nm (Liu et al., 2020a).

### 2.8 Determination of field yield and land equivalent ratio

To measure land area efficiency when intercropping, Willey and Osiru (1972) introduced the land equivalent ratio (LER) concept, which reflects the yield of two crops as intercrops and as a monocrop, which can be used to evaluate the suitability of two crops for intercrop cultivation. It is:


$$LER=pLER_{1}+pLER_{2}=\frac{Y_{1}}{M_{1}}+\frac{Y_{2}}{M_{2}}$$

where p LER is a partial land equivalent ratio, *Y_1_* and $Y_{2}$ are the yields of seed 1 and seed 2 in interplanting, *M_1_* and $M_{2}$ are the yields of seed 1 and seed 2 in single crop, respectively.

### 2.9 Data analysis

All statistical analyses were performed using various packages in R version 3.3.0 (Vermeulen et al., 2013), including ANOVA tests for multiple comparisons of soil microbial diversity, soil properties, active ingredient content, and plant biomass. Root secretion aroma diversity and evenness indices were compared among the groups by unpaired *t*-tests. When *p<* 0.05, the means of the groups were considered significantly different using the least significant difference (LSD). Principal component analysis (PCA), redundancy analysis (RDA), and heatmaps were generated using the “ggplot2” package in the R platform. PCA regroups all the metabolites originally identified linearly to form a new set of variables to determine differences between the groups of samples. To determine the proportion of variation in community structure explained by environmental factors, microbial community compositional components were determined by RDA, followed by variance decomposition analysis (VPA). Heatmaps were used to compare correlations between species abundance (OTUs) and various indicators, including environmental factors, plant growth parameters, and root base secretions.

## 3 Results

### 3.1 Effect of different planting methods on *S. miltiorrhiza* growth and development

In commercial trade, *S. miltiorrhiza* yield, quality, and appearance are very important for *Salvia* growers to achieve profitability. The *S. miltiorrhiza* fresh weight index can represent its yield, while hydrophilic and lipophilic components as well as root length and diameter indicate its quality and appearance (Liu et al., 2020a). Inter-root secretions between different plants had a significant effect on *Salvia* morphology (**Table 1**), and *Salvia* dry weight in the *Salvia*Sesame treatment increased by 3.21% compared to that in *Salvia*|Sesame when the plants were compared with and without the isolated treatment. *S. miltiorrhiza* fresh weight, dry weight, and root diameter were significantly decreased by inter-root secretions from maize, and all *Salvia* growth indices were decreased in *Salvia*Soybean and *Salvia*Maize compared to *Salvia*Sesame. Overall, inter-root secretions from sesame had the least effect on *S. miltiorrhiza* root development. The combinations *Salvia*Maize and *Salvia*Maize significantly inhibited growth, as *Salvia* fresh weight, dry weight, and root diameter decreased by 46.29%, 46.77%, and 27.88%, respectively.

**Table 1:** Single plant yield and agronomic traits of *Salvia* Tanshinone under different cropping patterns (mean ± S.E., *n* = 5).

Treatments	Fresh weight (g)	Dry weight (g)	Number of branches	Root length (cm)	Root width (mm)
*Salvia*Maize	105.00 ± 25.16d	38.93 ± 9.51c	12.60 ± 3.21	24.50 ± 5.32a	9.70 ± 2.16c
*Salvia*|Maize	195.50 ± 5.26c	73.14 ± 8.92b	13.60 ± 5.08	32.25 ± 3.59a	13.45 ± 1.48b
*Salvia*Soybean	227.50 ± 9.57bc	79.19 ± 2.88b	16.20 ± 4.66	30.95 ± 4.73a	12.01 ± 1.65b
*Salvia*|Soybean	255.00 ± 62.45ab	92.55 ± 23.84ab	14.00 ± 8.60	37.37 ± 14.67a	13.55 ± 1.24ab
*Salvia*Sesame	288.50 ± 53.75a	107.55 ± 16.19a	18.20 ± 7.11a	30.50 ± 5.26a	13.70 ± 1.63a
*Salvia*|Sesame	297.50 ± 37.75a	104.20 ± 20.04a	13.80 ± 3.27a	37.75 ± 15.28a	13.97 ± 0.95a

maize + *Salvia* completely isolated (*Salvia*|Maize), maize + *Salvia* without isolation (*Salvia*Maize), soybean + *Salvia* completely isolated (*Salvia*|Soybean), soybean + *Salvia* without isolation (*Salvia*Soybean), sesame + *Salvia* completely isolated (*Salvia*|Sesame), and sesame + *Salvia* without isolation (*Salvia*Sesame). Standard errors (S.E.). Boxes with various lowercase letters indicate significant differences between various regimes based on the least significant difference (LSD) test (*p*< 0.05).

**Figure 3:**
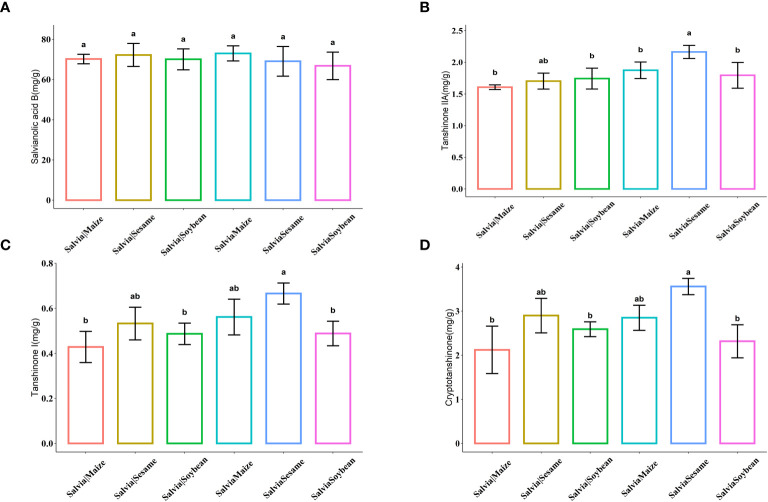
Histogram of active ingredient content of *Salvia* miltiorrhiza (*n* = 5). **(A)** Salvianolic acid B, **(B)** Tanshinone IIA, **(C)** Tanshinone I, and **(D)** Cryptotanshinone. Boxes with different lowercase letters indicate significant differences between various regimes based on the least significant difference (LSD) test (*p*< 0.05).

Active ingredient contents were compared under the six cultivation combinations (**Figure 3**). There were no significant differences in tanshinolic acid B. After *Salvia*Sesame treatment, *S. miltiorrhiza* tanshinones significantly increased, with the highest levels of Tanshinone IIA, Tanshinone I, and Cryptotanshinone, showing increases of 27.06%, 22.76%, and 26.41%, respectively, compared to *Salvia*|Sesame. Sesame and *Salvia* cultivation effectively increases accumulation of Tanshinone active ingredients.

### 3.2 Effect of different planting methods on soil properties

**Figure 4:**
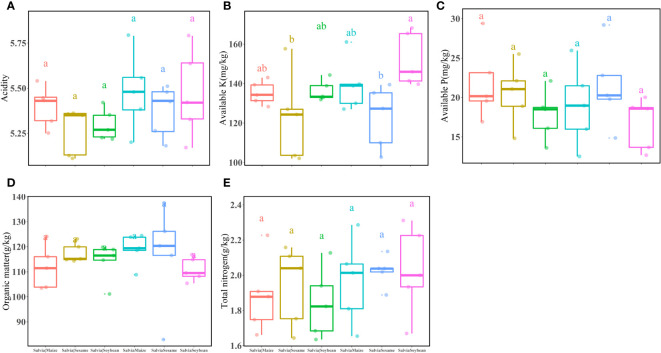
Inter-root soil factors under different cropping practices of *Salvia* and other crops (*n* = 3). **(A)** acidity, **(B)** available K, **(C)** available P, **(D)** organic matter, **(E)** total nitrogen. Boxes with different lowercase letters indicate significant differences between various regimes based on the LSD test (*p*< 0.05).

In the potted soil after each treatment group (*Salvia*|Soybean, *Salvia*Soybean, *Salvia*|Maize, *Salvia*Maize, *Salvia*|Sesame, and *Salvia*Sesame), there were no significant differences in acidity (**Figure 4A**), available P (**Figure 4C**), organic matter (**Figure 4D**), and total nitrogen (**Figure 4E**). The highest available K (**Figure 4B**) was found in *Salvia*Soybean (152.13 ± 13.69), which was significantly higher than available K in *Salvia*Sesame (122.94 ± 15.96), while there were no significant differences between plants with and without compartment treatments.

### 3.3 Effect of different planting methods on the *S. miltiorrhiza* inter-root bacterial community

Illumina detected a total of 1,121,526 clean reads generated from the 16S RNA gene in 18 samples, and a total of 8,654 OTUs were identified in all samples. The Chao1 index (a measure of α-diversity) was significantly lower for *Salvia*Soybean and *Salvia*Maize than for *Salvia*Sesame, with *Salvia*Soybean having the lowest (275.67 ± 83.28c) number of species (**Table 2**). The Shannon and Simpson indices indicated that bacterial diversity and homogeneity were typically better for all treatments, with greater variation for *Salvia*Soybean.

**Table 2:** Diversity indices of the inter-root microbial communities of *Salvia miltiorrhiza* under different cropping patterns (mean ± S.E., *n* = 3).

Treatments	ACE	Chao1	Simpson	Shannon
*Salvia*Maize	398.67 ± 28.57b	398.67 ± 28.57b	0.9930 ± 0.0010a	7.92 ± 0.22a
*Salvia*|Maize	532.71 ± 15.01a	532.67 ± 15.04a	0.9936 ± 0.0016a	8.21 ± 0.21a
*Salvia*Soybean	275.67 ± 83.28c	275.67 ± 83.28c	0.9420 ± 0.0317b	5.85 ± 1.01b
*Salvia*|Soybean	524.72 ± 39.22a	524.67 ± 39.31a	0.9925 ± 0.0012a	8.14 ± 0.07a
*Salvia*Sesame	580.67 ± 41.06a	580.67 ± 41.06a	0.9947 ± 0.0011a	8.46 ± 0.15a
*Salvia*|Sesame	571.38 ± 58.93a	571.33 ± 58.96a	0.9937 ± 0.0008a	8.36 ± 0.17a

This shows the richness (ACE, Chao1) and diversity (Simpson, Shannon) of the bacterial community. Standard errors (S.E.). Boxes with various lowercase letters indicate significant differences between various regimes based on the least significant difference (LSD) test (*p*< 0.05).

**Figure 5:**
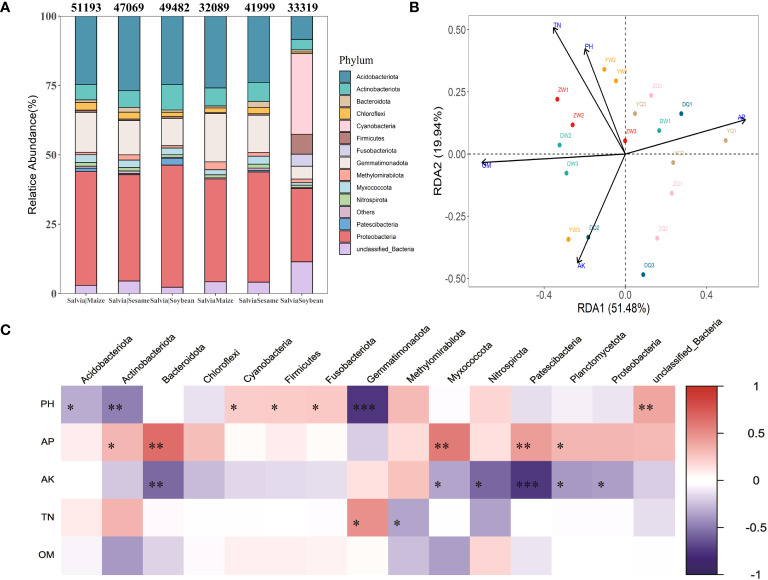
Distribution and composition of bacterial communities at the gate level under different cropping patterns (*n* = 3). **(A)** Relative abundance of staple microbial taxa on phylum level across all samples detected by 16S rRNA gene sequencing. The numbers above the bars indicate the number of microorganisms classified at the phylum level according to the sequence composition of feature. **(B)** Redundancy analysis of soil microbial communities and environmental parameters (RDA); arrows present the magnitude and direction of environmental factors associated with bacterial community structure. **(C)** Heatmap of soil environmental factors and phylum level bacterial correlations. Negative correlations and positive correlations are represented in blue and red. * indicates 0.01< *p* ≤ 0.05, ** indicates 0.001< *p* ≤ 0.01, *** indicates *p* ≤ 0.001.

Additionally, there were differences in bacterial community structure after the treatment in each group. The highest abundance at bacterial phylum level was found in Proteobacteria (26.41%–44.04%), Acidobacteriota (8.37%–26.88%), Gemmatimonadota (4.62%–17.40%), Actinobacteriota (3.78%–9.13%), and unclassified_Bacteria (2.28%–11.44%) (**Figure 5A**). Cyanobacteria and Fusobacteriota significantly increased in the *Salvia*Soybean treatment, accounting for 29.11% and 4.34% of total community abundance, respectively, while both did not exceed 0.1% in any other treatment. *Salvia*Maize and *Salvia*Soybean bacterial community abundance and number showed a significant decrease compared to *Salvia*|Maize and *Salvia*|Soybean, while *Salvia*Sesame and *Salvia*|Sesame bacterial community structure did not. There was no significant difference in the structure of *Salvia*Sesame and *Salvia*|Sesame bacterial communities. An RDA was performed to accurately assess the relative effects of soil physicochemical properties (potential of hydrogen, organic matter, total nitrogen, available P, and available K) on the abundance and diversity of *S. miltiorrhiza* inter-rhizosphere bacterial communities. The first two RDA components explained 51.48% and 19.94% of the overall variation, respectively. Furthermore, potential of hydrogen, available P, available nitrogen, total nitrogen, and organic matter appeared to play a crucial role in building bacterial communities (**Figure 5B**). There was a negative correlation between organic matter and RDA1 (70.64%), a direct correlation between available P and RDA1 (59.13%), and a maximum contribution of 50.81% from total nitrogen to RDA2. This indicates that organic matter, available P, and total nitrogen remain the key environmental factors affecting *Salvia* inter-root microorganism abundance. The soil environmental factor and microbial communities correlation analysis revealed that the potential of hydrogen and Gemmatimonadota were significantly negatively correlated (*r* ^2^ = 0.74; *p<* 0.001), and available K and Patescibacteria were significantly negatively correlated (*r* ^2^ = 0.74; *p<* 0.001) (**Figure 5C**).

### 3.4 Effect of different planting methods on *S. miltiorrhiza* inter-root secretions

**Figure 6:**
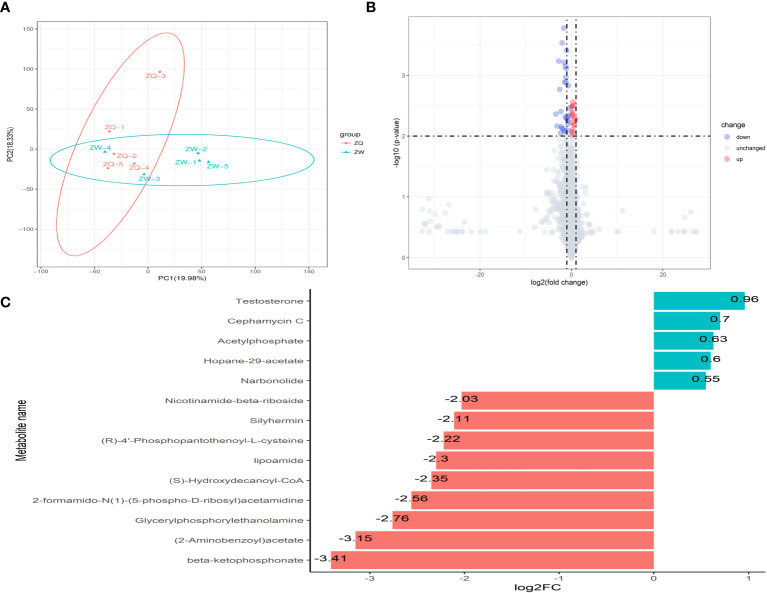
Analysis of inter-root secretion differences in *Salvia* (*n* = 3). **(A)** Principal component analysis (PCA) of inter-root secretion between *Salvia*|Sesame, and *Salvia*Sesame. **(B)** Volcano plot depicting inter-root secretions between *Salvia*Sesame and *Salvia*Soybean. **(C)** Fold change in the expression of inter-root metabolites of *Salvia miltiorrhiza* between *Salvia*Sesame and *Salvia*Soybean. **(B)** Each point in the graph represents a metabolite; the horizontal coordinate represents the log2 value of the fold difference of a metabolite between the two samples; the vertical coordinate represents the log10 value of the *p*-value. Red points represent upregulated differentially expressed metabolites, blue points represent downregulated differentially expressed metabolites, and gray points represent metabolites that were detected but did not meet the filtering parameters. **(C)** FC (fold change).

**Figure 7:**
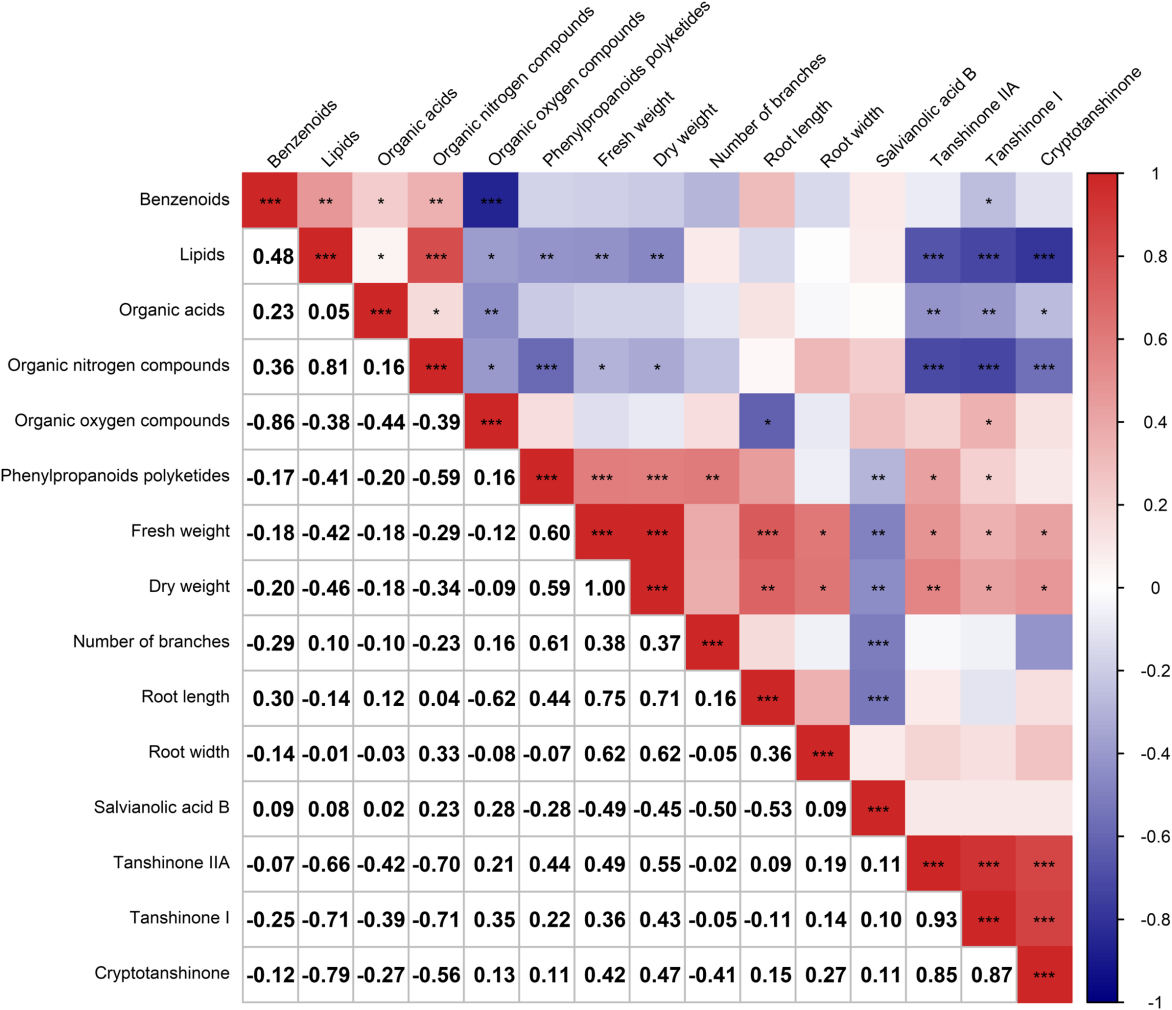
Correlation heatmap depicting the relationship between the six major root secretions and *Salvia miltiorrhiza* growth and development (*n* = 3). Negative correlations and positive correlations are represented in blue and red, respectively. * indicates 0.01< *p* ≤ 0.05, ** indicates 0.001< *p* ≤ 0.01, *** indicates *p* ≤ 0.001.

After analyzing the inter-root microbial community and agronomic trait indicators of *Salvia divinorum*, we found that sesame was the most promising plant to grow with it. The relationship between sesame and *S. miltiorrhiza* inter-root secretions was further analyzed by PCA, and there were similar metabolites between *Salvia*Sesame and *Salvia*|Sesame (**Figure 6A**). The *Salvia divinorum* inter-root microbial community was analyzed when the three crops were not isolated, and the lowest microbial community α-diversity was under *Salvia*Soybean treatment, while the highest microorganism abundance and number were found under *Salvia*Sesame treatment. This may be due to different crops resulting in different inter-root *S. miltiorrhiza* feedbacks. Analysis of *Salvia*Soybean and *Salvia*Sesame inter-root secretions revealed that 30 were significantly reduced in the *Salvia*Soybean treatment (*p*< 0.05 and VIP > 1.5) (**Figure 6B**), of which 18 belonged to the Human Metabolome Database (HMDB), with lipids and lipid-like compounds, nucleosides and analogs, organic acids and derivatives, organic oxides, and heterocyclic compounds accounting for 4.05%, 26.56%, 2.84%, 15.20%, and 8.27%, respectively. The two lowest *p*-value secretions screened, D-Sedoheptulose 7-phosphate and Nicotinamide-beta-riboside, both belong to the organic oxide class, and comparing the amounts of these two metabolites in *Salvia*Soybean and *Salvia*Sesame, the difference multiplicity (fold change) was −1.63746 and -2.02996 (**Figure 6C**), respectively. The correlation heatmap between *S. miltiorrhiza* yield, quality, and active ingredients showed that lipids were significantly and negatively correlated with yield (fresh weight and dry weight) and quality (Tanshinone IIA, Tanshinone I, and Cryptotanshinone) (**Figure 7**).

### 3.5 Relationships between microbial communities and *Salvia* root secretions, growth, and development

**Figure 8:**
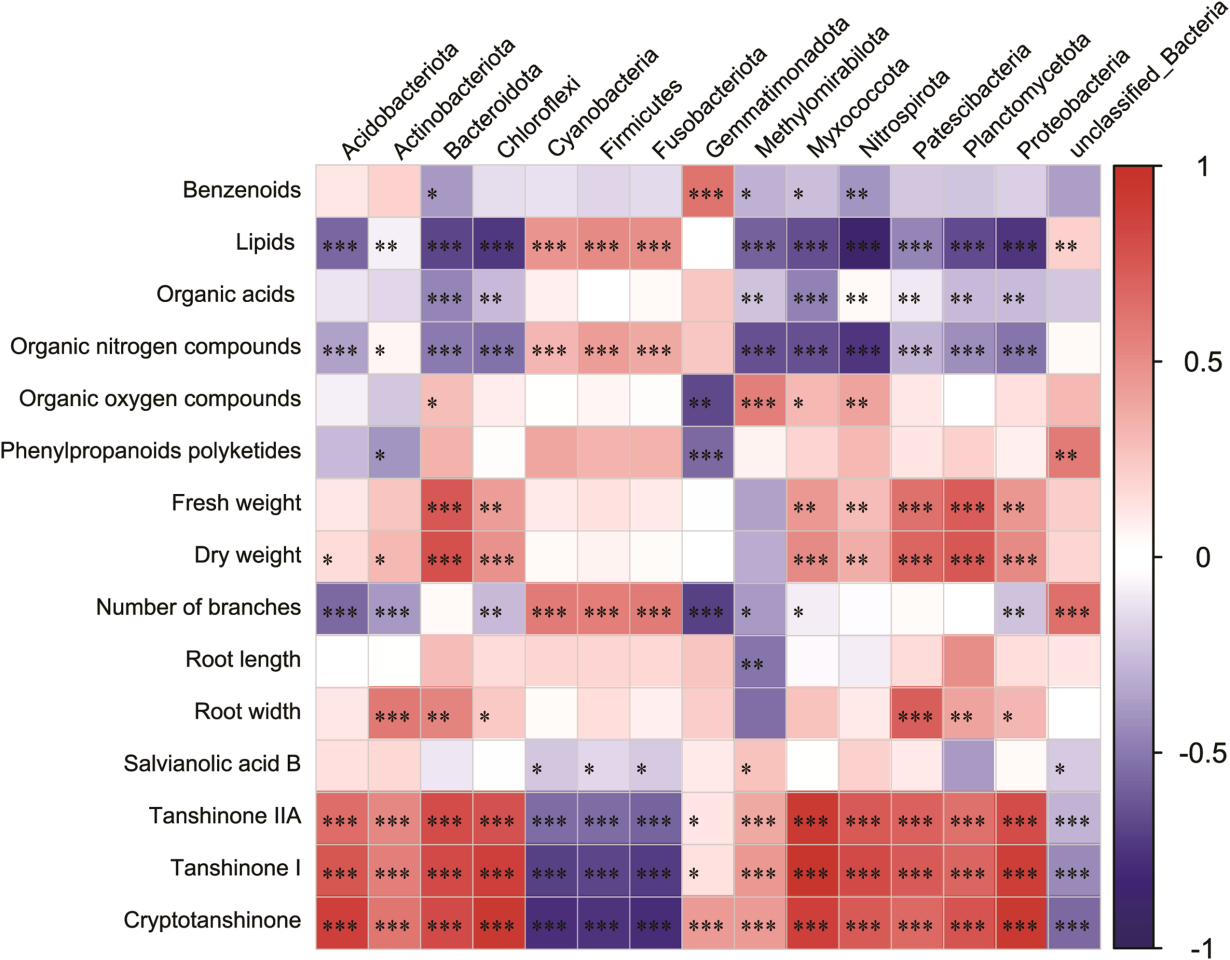
Correlation heatmap analysis of the association between the relative abundance of bacterial phyla and major inter-root secretions, and *Salvia miltiorrhiza* growth and development, including the relative richness (*n* = 3) of the top 15 microbial phyla. Negative correlations and positive correlations are represented by blue color and red color, respectively. Horizontal ordinate represents bacterial community abundance information, and vertical ordinate represents inter-root secretion and *Salvia miltiorrhiza* growth and development indicators. * indicates 0.01< *p* ≤ 0.05, ** indicates 0.001< *p* ≤ 0.01, *** indicates *p* ≤ 0.001.

To accurately assess the effects of microbial communities on root secretions and *S. miltiorrhiza* growth and development, we investigated the relationships between the main secretions (lipids, nucleosides and analogs, organic acids and derivatives, organic oxygen compounds, organoheterocyclic compounds, phenylpropanoids, and polyketides), *Salvia* basic growth and developmental indicators (fresh weight, dry weight, and number of nucleosides), *Salvia* active constituents (Salvianolic acid B, Tanshinone IIA, Tanshinone I, and Cryptotanshinone), and the main microbial communities. Lipids and lipid-like molecules and Bacteroidota, Chloroflexi, Nitrospirota, Planctomycetota, and Proteobacteria were significantly negatively correlated (*p*< 0.05) (**Figure 8**). Organic nitrogen compounds, root width, dry weight, and Patescibacteria all showed significant positive correlations (*p*< 0.05). Fresh weight, dry weight and Bacteroidota, Planctomycetota were significantly positively correlated with each other (*p*< 0.05). Interestingly, Bacteroidota, Planctomycetota, and Patescibacteria were all significantly positively correlated with each other (*p*< 0.05).

### 3.6 Field experiment yield and land equivalent ratio

Compound planting of different plants and *Salvia* led to differences in harvest yield and LERs. Averaging the data from three replications, *S. miltiorrhiza* yield after intercropping maize, soybean, and sesame was 9,817.20 ± 322.20, 14,817.451,950.15, and 15,739.65 ± 3,061.20 kg/hm^2^, respectively, while monocropping maize, soybean, sesame, and *Salvia* yields were 17,385.001,967.7, 3,432.45 ± 488.85, 1,800.15361.20, and 16,470.00 ± 3,616.80 kg/hm^2^, respectively. The lowest *Salvia* yield was after intercropping with maize, where it was reduced by 40.44%. *Salvia* yield was reduced by 10.11% and 4.52% when intercropped with soybean and sesame, respectively. The highest LER (1.82) was achieved with sesame and *Salvia* intercropping, with *pLER_1_* and $pLER_{2}$ of 0.96 and 0.86, respectively, and the lowest LER of 1.36 for soybean and *Salvia* intercropping. Field experiments verified that sesame and *Salvia* are a good intercropping combination.

## 4 Discussion

Compound planting patterns can effectively improve yield and quality of many medicinal plants, such as *Salvia* and *Angelica*, which are affected by successive crop barriers (Zhang et al., 2013; Liu et al., 2020a; Maitra et al., 2021). Compared to monocropping, Liu et al. (2018) showed that intercropping cultivation significantly enhanced *Salvia* root fresh weight, dry weight, and lipid-soluble component content. We showed that the combined planting of maize and *Salvia* reduced *Salvia* yield, a result consistent with previous studies (Lei et al., 2018); however, sesame and *Salvia* intercropping significantly increased the tanninone content of *Salvia* roots, while individual plant fresh and dry weight, root diameter, and branch number all increased, as did the LER (1.82) in field experiments. Many studies have demonstrated that soil factors are influenced by intercropping systems (Tang et al., 2020), e.g., hydrogen potential and available P significantly increased in both inter- and non-inter-rooted soils in the sugarcane–peanut intercropping system study group (Pang et al., 2021). In this study, there were no significant changes in potential of hydrogen, organic matter, available P, and total nitrogen, which may be due to the soil buffering effect, and/or the effects of plants on altering soil structure, which often takes several years to complete (Fierer, 2017).

Intercropping systems similarly cause changes in inter-root microbial community composition, which are mainly due to fertilization (Guo et al., 2020), crop type (Prommer et al., 2020), and changes associated with crop diversity, such as root secretions (Mommer et al., 2016) and soil environmental factors (Stefan et al., 2021). High-throughput sequencing of 16S rRNA is now an established approach (Bhakta et al., 2017) for deep exploration of soil microbial community composition and abundance. Li et al. (2022) found that Acidobacteriota and Proteobacteria were the dominant phyla in the maize intercropping system, and Proteobacteria relative abundance in the intercropped maize root soil increased significantly, primarily due to interspecific root interactions resulting in differences in bacterial community structure during intercropping. Meanwhile, Wang et al. (2019) showed that crop rotation and crop set could improve soil quality and inter-root bacterial diversity to some extent, and Symbiodinium relative abundance was higher in crop rotation and crop set than in continuous crop. In our experiment, Proteobacteria (26.41%–44.04%) and Acidobacteriota (8.37%–26.88%) were the dominant phyla, and interestingly, they (along with Salvia ketone content) were significantly and positively correlated with the fresh and dry weights of *Salvia* roots that were likewise positively correlated. Meanwhile, alpha diversity of the *S. miltiorrhiza* inter-root microbial community was highest in bacterial number and abundance when sesame and *S. miltiorrhiza* were grown in combination, while it was lowest when soybean and sesame were grown in combination. Thus, microbial community structure remains an important driver of *S. miltiorrhiza* root yield and quality.

The soil environment is the primary influencing factor in determining microbial community structure (Fierer, 2017). We found through RDA that soil environmental factors explained 51.48% and 19.94%, respectively, of the first two RDA components, which again confirmed previous studies. Available P, organic matter, and total nitrogen made the highest contribution, available P was the key factor affecting *S. miltiorrhiza* quality, and root Tanshinone IIA concentration was significantly and negatively correlated with soil effective phosphorus (Liang et al., 2021). Soil phosphorus content is also an important factor in soil bacterial community formation (Liu et al., 2020b). Additionally, total nitrogen is also considered the dominant factor in building microbial communities because it has key functions in cellular metabolic processes, such as energy metabolism, protein synthesis, and cell division (Rajan et al., 1991). Simultaneous analysis of soil environmental factors and microbial communities found that potential of hydrogen was negatively correlated with Gemmatimonadota, which is consistent with the findings of Guo et al. (2017).

In addition to inter-root secretions being a key factor affecting inter-root microorganisms (Wang et al., 2022), root secretion-induced autotoxin accumulation is also widely recognized as an important contributor to crop succession disorders (Xu et al., 2015). The study revealed correlations between inter-root secretion and *Salvia* microbial communities as well as the *Salvia* growth and development index. It was observed that lipids and lipid-like molecules had a significant and negative correlation with *Salvia* yield (fresh weight and dry weight) and quality (Tanshinone IIA, Tanshinone I, and Cryptotanshinone). Additionally, lipids and lipid-like molecules and Bacteroidota, Chloroflexi, Nitrospirota, Planctomycetota, and Proteobacteria were also significantly negatively correlated. In previous studies, different inter-root secretions had different effects on shaping microbial community structure (Li and Wu, 2018; Zhao et al., 2021). Therefore, controlling lipids and lipid-like molecule contents in soil may be an important way to improve *Salvia* root yield and quality and play an active role in shaping *Salvia* inter-root microbial structure.

## 5 Conclusions

The present study demonstrates that the complex cultivation of sesame and *Salvia* is not only a viable agricultural practice but also an effective method for optimizing land resource utilization. When sesame and *Salvia* are planted together, they can significantly increase *Salvia* tanshinone content. Furthermore, the results of our 16S rRNA sequencing analysis indicate that the number and abundance of inter-root microbial communities in *Salvia* are significantly higher when sesame and *Salvia* are planted together, compared to when *Salvia* is planted with either soybean or maize. Soil factors and inter-root secretions play important roles in shaping the *Salvia* inter-root microbial community. In particular, inter-root secretions had a significant influence on *Salvia miltiorrhiza* yield and quality.

## Data availability statement

The original contributions presented in the study are publicly available. This data can be found here: https://www.ncbi.nlm.nih.gov/bioproject under the accession number PRJNA992203.

## Author contributions

LZ and CZ designed the experiment. LZ wrote the paper and CZ revised the manuscript. ST, YZ, YY, FP, HL, CM, XW, YW, and ZX contributed to experiments and the acquisition of data. All authors contributed to the article and approved the submitted version.
